# Tented elevation with numerous tractions (TENT) technique to aid endoscopic submucosal dissection of a large cecal lesion

**DOI:** 10.1055/a-2208-6542

**Published:** 2024-01-09

**Authors:** Sujata May Hernaez Mansukhani, Yohei Minato, Tony He, Rosula Esther Castillo Sanchez, Ioannis Marakis, Shunya Takayanagi, Ken Ohata

**Affiliations:** 1 13635 Gastroenterology, NTT Medical Center Tokyo, Shinagawa-ku, Japan; 2 218384 Section of Gastroenterology and Digestive Endoscopy, Manila Doctors Hospital, Manila, Philippines; 3 13635 Gastroenterology, NTT Medical Center Tokyo, Shinagawa-ku, Japan; 4 60078 Gastroenterology, St Vincent's Hospital Melbourne Pty Ltd, Fitzroy, Australia; 5 280152 Gastroenterology, Hospital Nacional Cayetano Heredia, Lima, Peru; 6 69068 Endoscopy, Agios Savvas General Hospital Hellenic Institute Against Cancer, Athens, Greece

Colorectal endoscopic submucosal dissection (ESD) remains a challenging procedure and various traction methods to help decrease the risk of complications have been studied 1 2 3 . We present the tented elevation with numerous tractions (TENT) technique, an individually placed, multidirectional, multipoint, internal traction method for ESD of a large cecal lesion.

A 79-year-old woman who had been diagnosed with a 40-mm laterally spreading tumor (LST-G [nodular mixed]) in the cecum during a screening colonoscopy was referred for ESD ( Fig. 1 ).

**Fig. 1:**
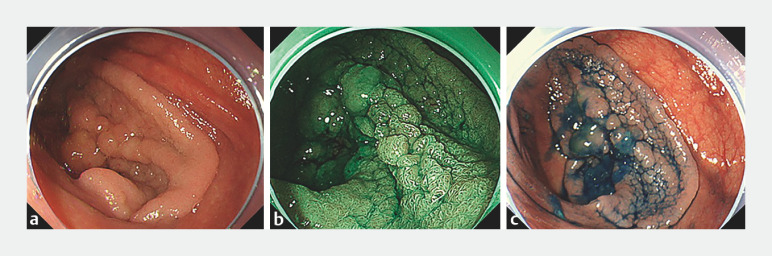
Endoscopic images showing a 40-mm laterally spreading tumor (granular, nodular mixed type) in the cecum under: **a** white light; **b** narrow-band imaging; **c** white light after flushing with indigo carmine.

After a partial mucosal incision and dissection had been performed, a single clip-band elastic traction device was deployed on the dissected mucosa and attached to the contralateral mucosa using a repositionable hemostatic clip ( Fig. 2 **a,b** ). Because of the size and location of the lesion, the dissection plane was still poorly visualized owing to collapse of the dissected mucosa onto the remaining undissected submucosal layer ( Fig. 2 **c,d** ). Using the TENT technique, five more clip-band elastic traction devices were individually attached to multiple points on the dissected mucosa and fixed in different directions to further lift the lesion and increase the visibility of the submucosal dissection plane ( Fig. 3 **a,b** ). With adequate tension having been achieved, the ESD knife was then positioned perpendicular to the vertically taut submucosal fibers, providing a safe plane for dissection ( Fig. 3 **c,d** ) *.* The total procedure time was 80 minutes. Complete resection was achieved with no complications ( Fig. 4 and Fig. 5 ; Video 1 ).

**Fig. 2:**
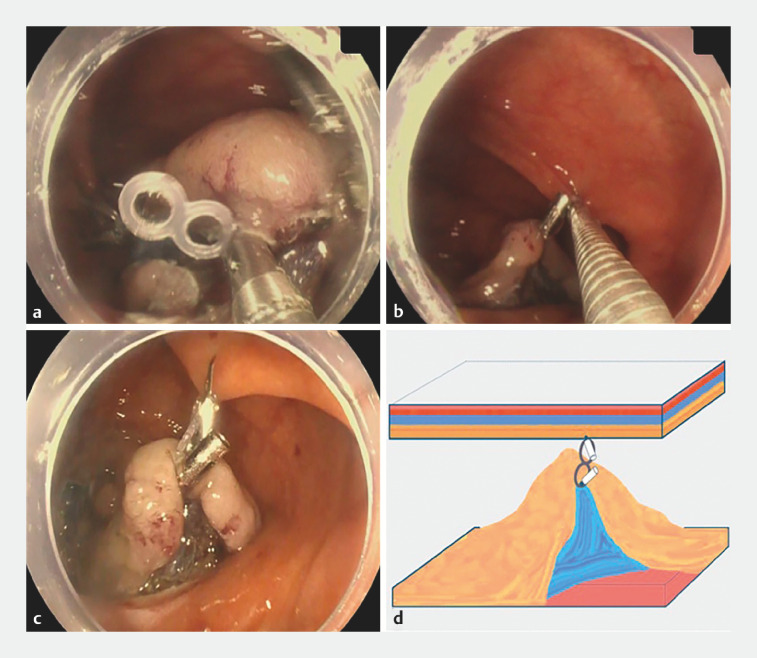
Images of traction using a single clip-band elastic traction device showing: **a,b** the traction device deployed between the dissected mucosa and submucosal layer and the contralateral mucosa using repositionable hemostatic clips; **c,d** subsequent collapse of the dissected mucosa onto the undissected lesion.

**Fig. 3:**
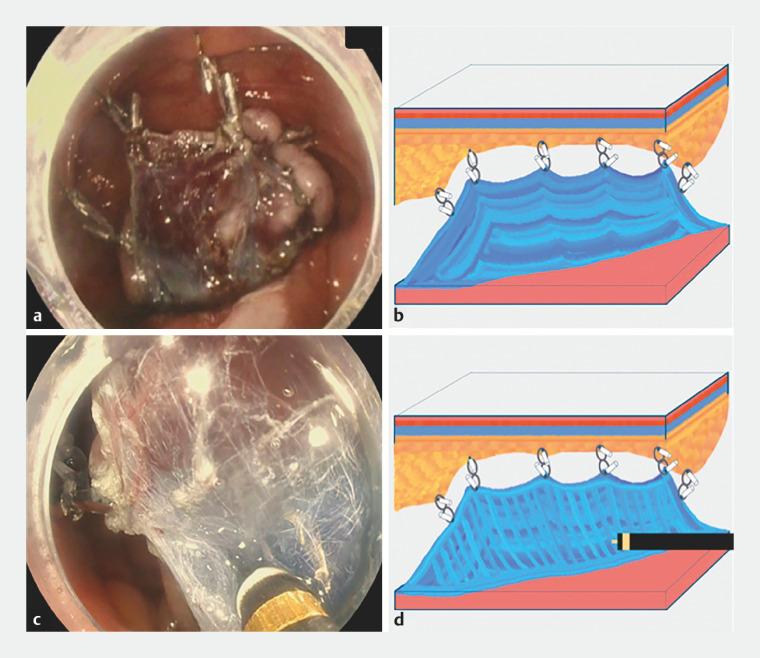
Images of the tented elevation with numerous tractions (TENT) technique showing: **a,b** several clip-band elastic traction devices deployed on multiple points of the dissected specimen and attached to different areas of the contralateral mucosa; **c,d** the perpendicular positioning of the endoscopic submucosal dissection knife against the submucosal layer after application of the TENT technique.

**Fig. 4:**
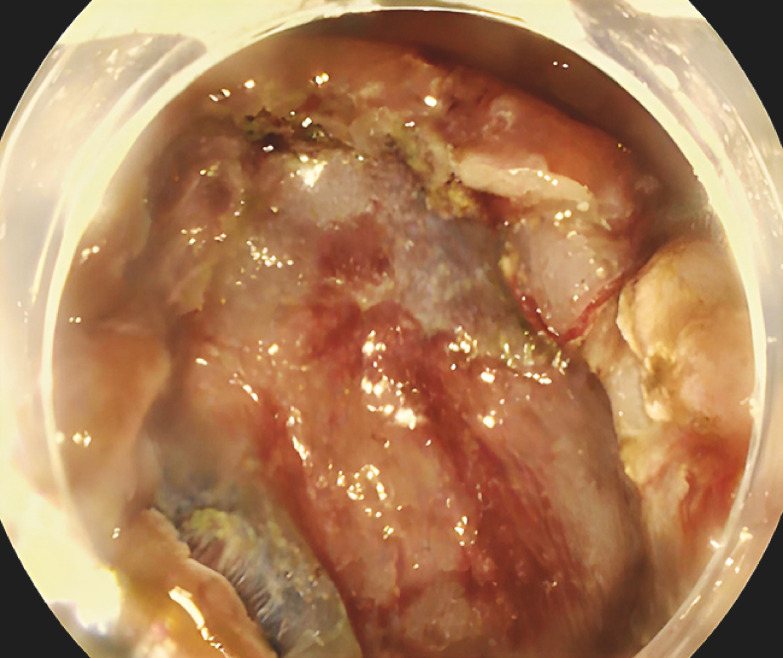
Endoscopic image showing the resection site, with no evidence of perforation or bleeding.

**Fig. 5:**
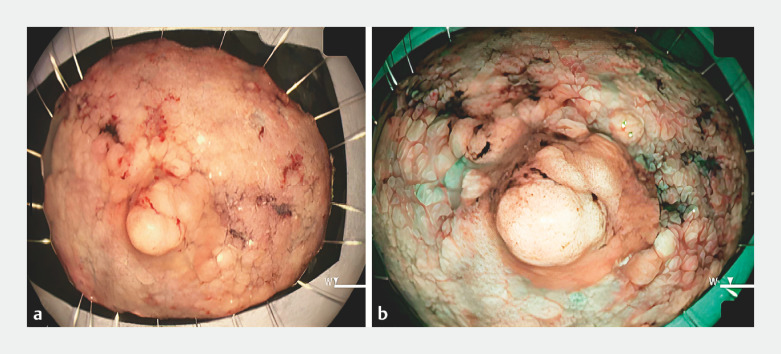
Macroscopic appearance of the resected specimen: **a** using white light; **b** after application of indigo carmine.

Demonstration of the tented elevation with numerous tractions (TENT) technique to resect a large cecal laterally spreading tumor.Video 1

To use the TENT technique effectively, it is necessary to apply traction, not only to the center of the lesion but also to both ends of the peeled area, to achieve a tent-like appearance. This is a modification of the conventional techniques, which apply traction only centrally or on a few random points, resulting in limited effectiveness because they do not adequately improve the visibility of the submucosal plane. Although the TENT technique requires more traction than is conventionally required, the clear visualization of the dissection plane results in more efficient, faster, and safer dissection.

Endoscopy_UCTN_Code_TTT_1AQ_2AD

## Notes

Endoscopy E-Videos https://eref.thieme.de/e-videos
*E-Videos* is an open access online section of the journal *Endoscopy* , reporting on interesting cases and new techniques in gastroenterological endoscopy. All papers include a high-quality video and are published with a Creative Commons CC-BY license. Endoscopy E-Videos qualify for HINARI discounts and waivers and eligibility is automatically checked during the submission process. We grant 100% waivers to articles whose corresponding authors are based in Group A countries and 50% waivers to those who are based in Group B countries as classified by Research4Life (see: https://www.research4life.org/access/eligibility/ ). This section has its own submission website at https://mc.manuscriptcentral.com/e-videos .
